# Information sharing for patient benefits: applying the Information Revolution to Telehealth in the UK

**Published:** 2012-06-15

**Authors:** Keith Naylor

**Affiliations:** Department of Health, UK

**Keywords:** information revolution, information sharing, standards, interoperability toolkit, ITK

## Abstract

Seamless, integrated care within and between organisations requires a massive change in the way information is currently shared and used, so that information is available to patients, clinicians and NHS staff, where and when they need it. Establishing telehealth and telecare services extend this challenge and these new services will need to be integrated into the wider Information Revolution that is driving change across the NHS.

Patients expect, and assume, that the NHS, social care and other organisations are joined up and understand their needs implicitly. We also need to remember that for some patients having access to ‘their’ information and being involved in decisions about their care is very important. However, all patients want to be treated with dignity, and have confidence that care they receive is safe and effective—information sharing plays a pivotal role in this.

The information sharing challenges faced by organisations are common, and call for national solutions, while the implementations need to address the realities of the local landscape. What is emerging is a technology landscape that supports a mixture of local and national information sharing arrangements that will provide NHS and other organisations with the ability to choose the most appropriate and efficient technology solution to meet their business needs.

Historically the local data sharing has been beset by a range of technical and commercial problems that mean that many solutions are not reusable across the NHS while national approaches have been inflexible and often costly to implement. A key part of the new information landscape is the Interoperability Toolkit initiative, which is providing a national framework, supported by an accreditation scheme, that addresses these local information sharing challenges.

There is much that is already available to address the information needs for large-scale telehealth and telecare services. This talk will review what is available and highlight ongoing work to support telehealth and telecare specific requirements, building on the work done by the Continua Health Alliance and in partnership with Newham for the Whole System Demonstrators.

